# Study on Improving the Processability and Properties of Mixed Polyolefin Post-Consumer Plastics for Piping Applications

**DOI:** 10.3390/polym13010071

**Published:** 2020-12-27

**Authors:** Emilia Garofalo, Luciano Di Maio, Paola Scarfato, Arianna Pietrosanto, Antonio Protopapa, Loredana Incarnato

**Affiliations:** 1 Department of Industrial Engineering, University of Salerno, 84084 Fisciano (SA), Italy; ldimaio@unisa.it (L.D.M.); pscarfato@unisa.it (P.S.); arpietrosanto@unisa.it (A.P.); lincarnato@unisa.it (L.I.); 2 COREPLA-Italian Consortium for the Collection and Recycling of Plastic Packages, Via del Vecchio Politecnico, 20121 Milano (MI), Italy; protopapa@ext.corepla.it

**Keywords:** flexible packaging, mixed polyolefin recyclate, piping applications, pipes stiffness

## Abstract

This study focuses on the upgrading strategies to make Fil-s (acronym for film-small), a polyolefin-based material coming from the mechanical recycling of post-consumer flexible packaging, fit for re-use in the piping sector. The effects of washing treatments (at cold and hot conditions) and the addition of an experimental compatibilizer on the chemical-physical properties of Fil-s were first assessed. The measurements of some key properties (density, melt flow index, flexural modulus, yield strength), for both Fil-s as such and the different developed Fil-s based systems, was also conducted in order to evaluate the suitability of this complex and challenging waste stream to replace virgin PE-based pipe and fitting products, in compliance to ASTM D3350 standard. The outcomes of the present work contributed to define a code, for each Fil-s system investigated, useful for identifying the level of their performance in piping applications. All the recyclates were extruded as pipes by using a pilot scale plant, but the process resulted more stable and continuous with the compatibilized Fil-s, as it was deducible from its flow properties. Moreover, the best mechanical performances were exhibited by the hot-washed Fil-s pipes, with an increase in pipe stiffness equal to 65% respect to the unwashed sample.

## 1. Introduction

A significant increment in the amount of post-consumer plastics waste sent for recycling can be deduced from the data collected over the last decade in Europe [1]. However, much more needs to be done to achieve greater circularity in the plastics industry, as fostered in the EU strategy on plastics [2,3]. First of all, in order to meet the European ambitious recycling targets for 2030, new opportunities must be created to valorize also the more complex and challenging plastic waste streams, such as post-consumer flexible packaging [4,5]. To this purpose, in the framework of the Circular Economy the well know principle of “Design for Recycling” [6,7] should be combined with the “Design from Recycling” approach, as suggested by Ragaert et al. in their works [8,9,10]. This latter methodology consists of making recycled polymers fit-for-use in designated products and/or redesigning products specifically for the recycled polymers. In other words, according with this approach, mechanically recycled polymers can be matched to potential new applications, following two design strategies: the first starts from the characteristics of the recycled material and then, based on these ones, defines the possible application; the second kicks off from the fundamental functional properties of the potential product and then selects the recyclate, using it as such or after proper upgrading steps.

The “Design from Recycling” approach must not necessarily be closed-loop to be valid. In fact, it may happen that it is possible to valorize a recycled material only for an application other than the original one. It might be the case of a post-consumer packaging material, that after the recycling process cannot generally be re-used in a food-contact application (due to contaminants’ migration issues), or because the resulting recycled material has composition and properties completely different compared with the virgin resin (for example, the mechanical recycling of multilayer films, made of different polymers types, results in polymer blends [11,12]).

Flexible packaging plays an essential role during its service life, by protecting the products (particularly food) throughout the supply chain and enabling a proper and safe delivery to the end-consumer. Moreover, it is also highly material-efficient and allows a reduced environmental impact (especially regarding the overall carbon footprint).

However, currently in Europe several flexible packaging product groups show low recycling rates. In particular, as stated in a recent report, delivered by the Plastics Recyclers Europe (PRE) organization, only 23% of PE flexible films (and 15% of all flexible films) were actually sent for recycling, with a portion that was lost in further sorting stages [13]. The major recycling challenges concern non-PE flexible packaging (such as PP monolayer films and multi-material/multilayer films) and, in general, films from households and agriculture. In fact, since these latter contain several contaminants, such as in pigments, other polymers, papers, and organics, it is more challenging to produce high quality recyclate [13].

In this context, some associations [14] were established and several R&D projects [15,16] were performed with the aim to build a circular economy for all flexible packaging, involving the whole supply chain, from polymer production and packaging manufacture to waste management and recycling. The hurdles in the mechanical recycling of flexible packaging were specifically addressed, suggesting areas of action to further close the loop. It was first evidenced that, in Europe, flexible packaging is often not considered sufficiently “valuable” by national Extended Producer Responsibility (EPR) schemes to be widely collected [16]. Moreover, in most European countries current plastic waste sorting processes treat flexible packaging as a potential contaminant for other sorted plastic fractions and are designed to extract it from the waste stream.

At present, in Italy all plastic packages are collected and the national sorting and recycling facilities are at the forefront for the treatment of flexible packaging waste, from which it is already possible to obtain an experimental flow of polyolefin-based recycled materials (made up by no less than 80% in weight of polyethylene). But, the next necessary step, to actually make flexible packaging relevant to the circular economy, consists in finding proper end-market applications for the secondary raw materials derived from it.

In recent years, recycled polyethylene (particularly high density polyethylene) has been used in several piping applications [17,18,19,20,21,22]. Some examples, already on the market, concern corrugated pipes for sewerage, drainpipes, cable ducts and irrigation pipes. In fact, due to properties such as ductility, chemical resistance, low thermal and electrical conductivity, polyethylene pipes are widely used in water and gas distribution, in agricultural irrigation and to convey different types of drains. Furthermore, compared to steel, polyethylene pipes are ten-times lighter and show other numerous advantages, including a ten-times lower environmental impact, which translates into a total energy saving of 38%, and a service life (as defined in the design phase) of about 50 years [23]. Using a recycled PE-based compound for the production of pipes allows to add another important feature to this type of product, i.e., the eco-sustainability.

In Italy, this interest is also supported through the “Plastic Second Life-Ecological Pipe” certification [24], a green label aimed at attesting the presence of recycled material (pre and/or post-consumer) in pipes composition, demonstrating the environmental sustainability of the products. The label introduces the concepts of quality and traceability also in the choice of recycled plastics.

The aim of this work was to evaluate the possibility of using Fil-s, a mixed polyolefin recycled material, obtained from the mechanical recycling of post-consumer flexible packaging of small size (<0.125 m^2^), for piping applications. Fil-s was supplied by COREPLA (the Italian Consortium for the Collection and Recycling of Plastic Packages) and, as extensively reported in our recent studies [25,26,27], it is primarily constituted of polyethylene (LLDPE and LDPE) and a lesser fraction of polypropylene, with traces of polar substances, both polymers (such as polyesters and polyamides) and low molecular weight contaminants. The critical issues, regarding the upgrading of Fil-s, essentially concern the incompatibility between the main polymer fractions inside the recycled material [25,26,27], its hygroscopicity [28] and bad smell.

The experimental plan, aimed at the production of pipes from Fil-s (as such and after proper upgrading steps) following the “Design from Recycling” approach, involved three fundamental phases:

(1) the characterization of Fil-s systems, which were developed to solve some of the main critical issues of this recyclate. In particular, the effects of washing treatments (both at cold and hot conditions) and the addition of an experimental compatibilizer on the spectroscopic, thermal and rheological properties of Fil-s were assessed;
(2) the determination of some key properties (density, melt flow index, flexural modulus, yield strength) associated with pipes performances (as defined in ASTM D3350 standard) for all the developed Fil-s systems;
(3) the pipes production by means of a pilot scale plant, starting from the heat-dried Fil-s based systems or the same undried materials, added with zeolite as desiccant, and finally, their mechanical characterization under crushing loads.

## 2. Materials and Methods

### 2.1. Materials

The recycled material used for this study was Fil-s (acronym for film-small) and was supplied as pellets by COREPLA, the Italian consortium for the collection and recycling of plastic packaging. It was obtained by the mechanical recycling of post-consumer flexible packaging of small size (<0.125 m^2^) and it is essentially made up of polyolefin fractions (primarily PE and smaller amounts of PP, ranging from 5% to 15% in weight). In particular, a Fil-s batch with a content of polypropylene of ca 5 wt% was used in the present study. COREPLA also supplied other two Fil-s lots that were subjected to washing treatments with caustic soda, both at cold (Cold Washed Fil-s, CW Fil-s) and hot conditions (at 85 °C for 10 min, Hot Washed Fil-s, HW Fil-s). The aim of these treatments was to reduce the levels of inks, adhesives, organic contamination, odors and microorganisms, improving the suitability of the recycled material for a wider set of outputs.

The proprietary experimental additive, grafted with maleic anhydride (MAH) and consisting of a PE/PP blend (with a content of PP lower than 5 wt%), was supplied by the company Auserpolimeri (Lucca, Italy), specialized in the continuous reactive extrusion with MAH of different polymers. The content of maleic anhydride inside the compatibilizer (denoted as COMP), was determined by means of a non-aqueous back-titration procedure [26] and resulted equal to about 0.5 wt%.

The amount of compatibilizing agent, added inside Fil-s and tuned in order to obtain the best increment in the mechanical performances of the recyclate, was set at 5% in weight.

The synthetic zeolite 4A, purchased from Honeywell Fluka (Modena, Italy), was used as desiccant during Fil-s extrusion and its main properties (according to the supplier) are summarized in Table 1. In order to remove the adsorbed water vapor or other volatiles, the zeolite was dehydrated at 300 °C for 16 h before use [29].

The preparation of the blend Fil-s+COMP was conducted using a twin-screw extruder (Dr. Collin GmbH–model ZK 25-48D, Maitenbeth, Germany) with co-rotating intermeshing screws (D_screw_ = 25 mm, L/D = 42). Before processing, the materials were dried in a vacuum oven at 70 °C for 18 h. Fil-s pellets were mechanically mixed with 5 wt% of the compatibilizer and the blend was fed into the extruder hopper by making use of a volumetric feeder. A screw speed of 100 rpm and temperatures of 190 °C, along the barrel, and 160 °C, at the die, were used. The extruded materials were cooled in a water bath and, then, pelletized.

### 2.2. Ribbons and Pipes Production

Fil-s based systems were extruded using a Do-Corder 330 instrument (Brabender Messtechnik GmbH, Duisburg, Germany) single-screw extruder (D_screw_ = 20 mm and L/D = 20), equipped with a flat die (20 mm × 1 mm). Before processing, the materials were dried in a vacuum oven at 70 °C for 18 h. A screw speed of 20 rpm and a temperature profile of 165-170-160 °C from hopper to die were imposed. The extruded ribbons were air cooled on a conveyor belt, whose speed was 0.5 m/min. The distance between the conveyor belt and the extruder head was approximately 10 cm.

The same single screw extruder, equipped with an annular die (D_est_ = 10 mm and D_int_ = 8 mm), was used for pipes extrusion, setting also the same operative conditions (thermal profile and screw speed) used for the ribbon production. A compressed air inlet is provided near the extrusion head to prevent the tube from collapsing on itself. Then, the pipe extrudate enters a calibrator whereby the use of vacuum fixes the outside diameter, followed by a water bath where cooling takes place. A take-up device constantly pulls on the pipe.

The pipes production was conducted using both the previously dried recyclates (in a vacuum oven at 70 °C for 18 h) and the not-dehumidified ones, but added with zeolite. The moisture contents for the heat-dried Fil-s systems and the corresponding samples, conditioned at a temperature of 25 °C and humidity of 75%, are reported in Table 2. The idea was to assess the effectiveness of zeolite as desiccant, by adding it during the pipes’ extrusion of the different Fil-s based systems, which were previously conditioned at more drastic RH values than the standard ambient conditions, so considering the worst case.

The pipes, produced with the different Fil-s-based systems, showed similar dimensions, i.e., an external diameter of 6 mm and a thickness of 0.6 mm.

### 2.3. Characterization Techniques

#### 2.3.1. Physical Properties

Differential Scanning Calorimetry (DSC) analyses were carried out using a DSC30 Mettler calorimeter (Mettler-Toledo International Inc., Columbus, OH, USA) and performing the following thermal cycle: a first heating at 10 °C/min from 0 °C to 250 °C; an isotherm at 250 °C for 5 min to melt the residual crystals, remove the thermo-mechanical history and eventual moisture residues; a cooling to 0 °C and a re-heating to 250 °C at the same scan rate. A value of ΔH_m_ = 293 J/g [30] was used as the reference melting enthalpy for 100% crystalline PE.

The AQUATRAC-3E moisture analyser (Brabender Messtechnik GmbH) was used to evaluate the moisture content in the samples before extrusion. The instrument is based on a chemical operating principle according to the standard ISO 15512.

The density measurements were carried out as stated in ASTM D792 standard, using an analytical balance (model BC, ORMA, Milan, Italy) and ethanol as an auxiliary liquid, whose density was calculated (ρ_f_ = 0.78377 g/cm^3^) at the test temperature of T = 26.5 °C. The density of the samples was determined by Equation (1):

(1) $$\rho =0.0012+\frac{Wa\left(\rho_{f}-0.0012\right)}{0.99983G}$$

where *W_a_* is the sample weight [g], *W_f_* is the weight of the immersion liquid [g] and *G* is calculated as (*W_a_-W_f_*) [g].

#### 2.3.2. Rheological Properties

The evaluation of the Melt Flow Index (MFI) values for all Fil-s based systems was carried out with the Melt Flow Junior meter (ITW Test and Measurement Italia-Instron Ceast Division, Pianezza, Italy). The measurements were performed, according with ASTM D1238 standard, at T = 190 °C and using a weight of 2.16 kg.

Rheological experiments in oscillatory mode were conducted with a rotational rheometer ARES (Rheometric Scientific, New Castle, DE, USA) under a nitrogen atmosphere. The tests were performed at 190 °C, in an angular frequency range from 0.1 to 100 rad/s, using 25 mm diameter parallel plates. A strain amplitude of 1% was proven to ensure linear viscoelasticity during the dynamic rheological measurements.

Rheological tests in elongational mode were performed by using a Sentmanat Extensional Rheometer (SER) [31,32]. This instrument (model SER-HV-A01, manufactured by Xpansion Instruments, Tallmadge, OH, USA) was designed for use as a detachable tool on ARES. Samples for testing were prepared by compression moulding, using a preheated hydraulic press, and cut into small rectangles with the following dimensions: thickness, width and length of 0.8 mm × 6 mm × 15 mm, respectively, which were specifically chosen within the recommended ranges of sample dimensions for optimum instrument performance [31]. The elongational tests were performed at 170 °C and different Hencky strain rates (0.5, 1, and 10 s^−1^), until the maximum achievable Hencky strain (equal to 3.8).

#### 2.3.3. Mechanical Properties

Tensile mechanical tests were performed, according to ASTM D882, by means of a CMT4000 Series dynamometer (SANS, Shenzhen, China). The specimens, cut from the extruded ribbons (50.8 mm long, 12.7 mm wide), were submitted to a crosshead speed of 5 mm/min to measure the Young’s modulus and 500 mm/min to determine the mechanical properties at break. A load cell of 1 KN was used to perform the tests.

All Fil-s based systems were also submitted to mechanical bending tests according to ASTM D790 standard. In particular, since all recycled specimens neither yield nor break before the 5% strain limit, the flexural tests were conducted following the procedure B of the standard. The samples, cut from the extruded ribbons were and tested flatwise on a 25.4 mm support span. The same CMT4000 Series dynamometer, equipped with flexural clamps and a load cell of 100 N, was used for the measurements, setting a crosshead speed of 7.5 mm/min, as calculated by Equation (2):

(2) $$R=\frac{ZL^{2}}{6d}$$

where *R* is the rate of crosshead motion [mm/min], *L* is the support span [mm], *d* is the depth of beam [mm], and *Z* is the rate of straining of the outer fibre [1/min], which has to be set equal to 0.01 if procedure B of the standard is followed.

All the extruded Fil-s pipes were characterized by parallel-plate loading tests according to the standard ASTM D2412. For these measurements the CMT4000 Series dynamometer was equipped with two parallel steel bearing plates and a load cell of 5 KN. The tests were carried out with a crosshead speed of 12.5 mm/min on pieces of pipe 100 mm long. The main parameter determined was the so-called pipe stiffness, calculated as the ratio between the force applied to the specimen per unit of length and a defined deflection value, Δ*y*, this latter representing the measured change of the inside diameter in the direction of load application. All the mechanical data were mediated on ten samples for each system analysed.

## 3. Results and Discussion

### 3.1. Characterization of Fil-s Based sysTems: Effect of the Washing Treatments

**Figure 1:**
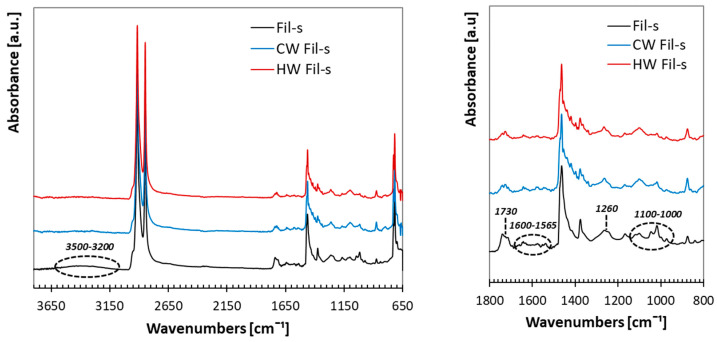
Comparison between the FTIR/ATR spectra of Cold Washed, Hot Washed and unwashed Fil-s. The peaks highlighted in the spectrum of Fil-s denote the presence of polar groups.

Both the washed Fil-s batches (cold CW and hot HW Fil-s) were characterized by spectroscopic (FTIR/ATR), thermal (DSC) and dynamic rheological tests. The obtained results were compared with the corresponding data of the unwashed Fil-s, in order to highlight eventual changes in the recyclate physical-chemical properties due to the washing processes. The FTIR/ATR spectra for the washed and unwashed Fil-s specimens are reported in Figure 1.

As extensively stated in our previous works [25,26,27], in addition to the main adsorption bands of polyethylene and polypropylene, other peaks (the ones highlighted in Figure 1) can also be detected in all the samples. These latter signals point out the presence of polar contaminants, both traces of polymers, such as polyamide or polyester, and low molecular weights materials. However, some differences in the intensities of these vibrational bands can be observed comparing the spectra of the recyclates, subjected or not to the washing phase. In particular, the broad band in the wavenumber range of 3500–3200 cm^−1^, which is characteristic for stretching vibrations of O-H and N-H bonds, disappears in CW and HW Fil-s. With regards the peak at 1730 cm^−1^, assigned to C=O stretching vibrations of the carboxylic acid group, it can be still observed in the washed samples, even if it is more pronounced for the unwashed Fil-s. The same comment can be done about the adsorption bands in the wavenumber range of 1600–1565 cm^−1^, assigned to the stretching vibrations of conjugated (C = C) bond and -NH group, which are less intense in the HW Fil-s spectrum. Conversely, the absorption peak at 1260 cm^−1^, characteristic of C-O stretching vibrations, is clearly visible in all the samples. As suggested by A. Gala et al. [12], who performed an extensive characterization of post-consumer plastic film recovered from mixed municipal solid waste, this signal is consistent with the presence of phenolic compounds used as polymer additives, such as antioxidants. Moreover, the same authors attribute the peaks in the region of 1100–1000 cm^−1^, also observed for all Fil-s based systems, to the stretching vibrations of silica (Si-O).

The main thermal parameters for the unwashed and washed Fil-s samples were also measured and the results are summarized in Table 3. In particular, as extensively reported in our previous papers on Fil-s [25,26,27], two melting peaks are exhibited by this recyclate. The first multiple peak can be attributed to the melting transition of the polyethylene phase in the sample, with a shoulder at lower temperature (T_mPE,1_ ≅ 110 °C), related to the presence of LDPE, and a more pronounced peak at higher temperature (T_mPE,2_ ≅ 123 °C), ascribable to LLDPE. A further melting peak at T_mPP_ ≅ 161 °C is related to the PP fraction of Fil-s.

No significant differences were evidenced in the melting temperatures of Fil-s, following the washing treatments, while it can be observed a relevant increment in the crystallinity degree for the polyethylene fraction (X_cPE_) of the washed Fil-s samples, particularly pronounced in the case of HW Fil-s. This outcome may be reasonable attributed to the removal of low molecular weight substances from the recyclate due to the washing step, as also suggested from the spectroscopic results.

**Figure 2:**
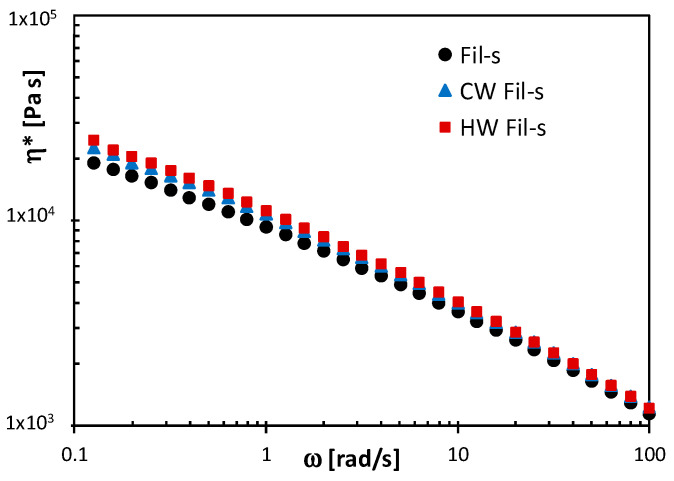
Comparison between the complex viscosity plots of the unwashed, Cold Washed and Hot Washed Fil-s samples.

The dynamic viscosity data, reported in Figure 2 for the washed and unwashed Fil-s specimens, further support this interpretation. In fact, in the whole frequency range analysed, the complex viscosity (η*) plots for both CW and HW Fil-s, which show an almost overlapping trend, shift towards higher η* values than the ones of the unwashed Fil-s sample. In other words, the contaminants with low molecular weight, which were removed from the recyclates by the washing step, act as plasticizers in the unwashed specimen.

On the basis of these results, degradation phenomena of the recyclate due to the washing treatments can be reasonably excluded.

### 3.2. Characterization of Fil-s Based Systems: Effect of the Addition of a Compatibilizing Agent

**Figure 3:**
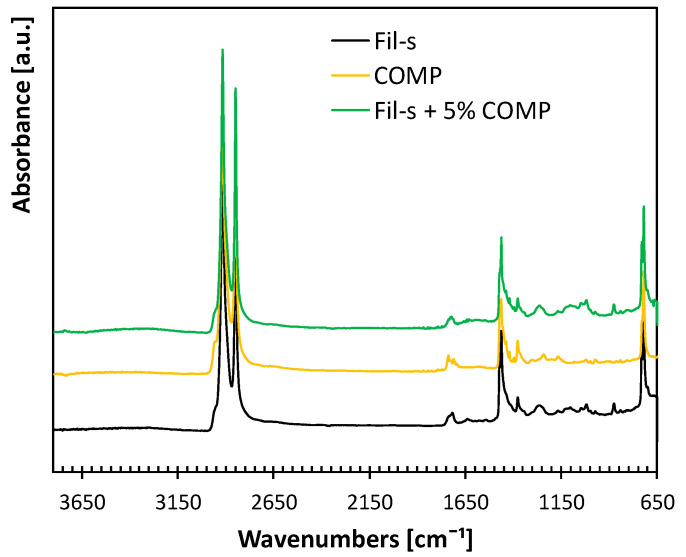
Comparison between the FTIR/ATR spectra of Fil-s, COMP and Fil-s + 5%COMP.

The FT-IR/ATR spectra of the neat Fil-s, the compatibilizer and their blend at 5 wt% of COMP are compared in Figure 3.

In particular, the FT-IR spectra assigned to Fil-s and COMP are very similar. In particular, the compatibilizer shows the typical absorption bands of PE (CH_2_ stretching vibrations at 2900–2800 cm^−1^) and PP (CH_3_ scissoring at 1376 cm^−1^), accompanied by the additional signal at 1730 cm^−1^, assigned to C = O stretching vibrations of carboxylic acid group and arising from its functionalization with maleic anhydride. As expected, the blend Fil-s + 5%COMP shows the signals of both its constituents, with no significant shifts in their position.

**Figure 4:**
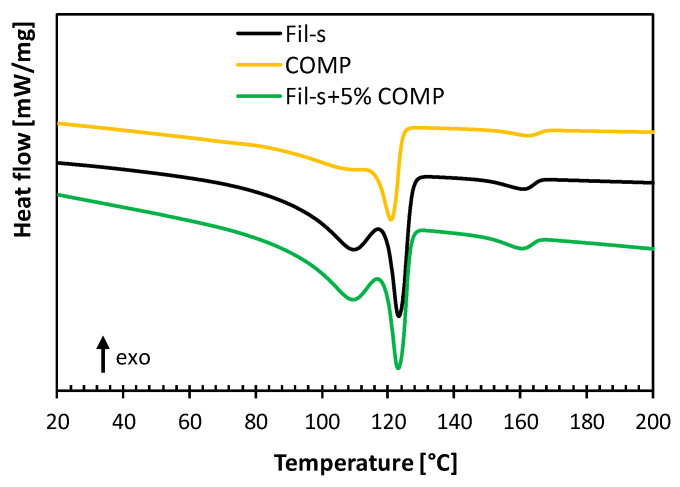
Comparison of the second heating thermograms of Fil-s, COMP and Fil-s + 5%COMP.

The second heating thermograms, reported in Figure 4, confirm the similar polymeric composition of COMP respect to Fil-s. Moreover, the addition of the compatibilizer at 5wt% inside Fil-s determines no significant change in both the melting temperatures and only a slight decrease of the crystallinity degree, which for the polyethylene fraction of the recyclate goes from 35% for the neat Fil-s to 33% for the compatibilized one.

**Figure 5:**
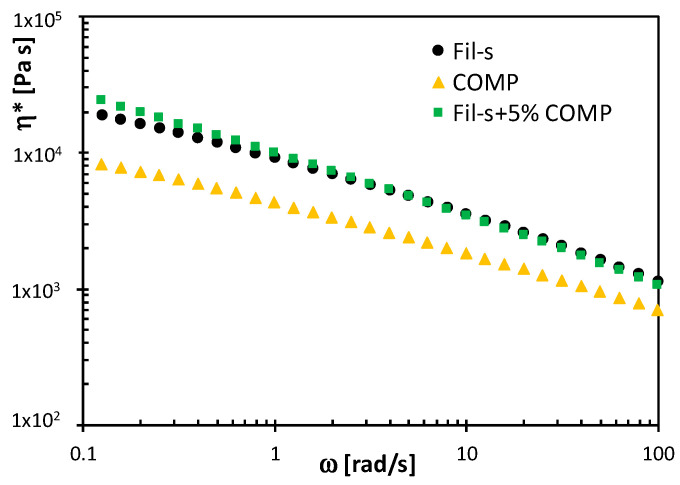
Comparison between the complex viscosity plots of Fil-s, COMP and Fil-s + 5%COMP.

The dynamic shear viscosity plots (η*) for Fil-s, the compatibilizing agent and their blend are compared in Figure 5.

In the whole frequency range investigated, the compatibilizer exhibits η* values significantly lower compared with Fil-s, and its addition inside the recyclate makes more pronounced its shear thinning behaviour, evidencing for the compatibilized material an even better processability by extrusion. The inter-polymer (both PE-PE or PP-PP and PE-PP) polar interactions between anhydride groups in the compatibilized Fil-s contribute to this outcome [26]. However, a similar effect on the complex viscosity could also be attributed to an increase of cross-linking and/or branching degree in the recyclate due to COMP addition [33].

**Figure 6:**
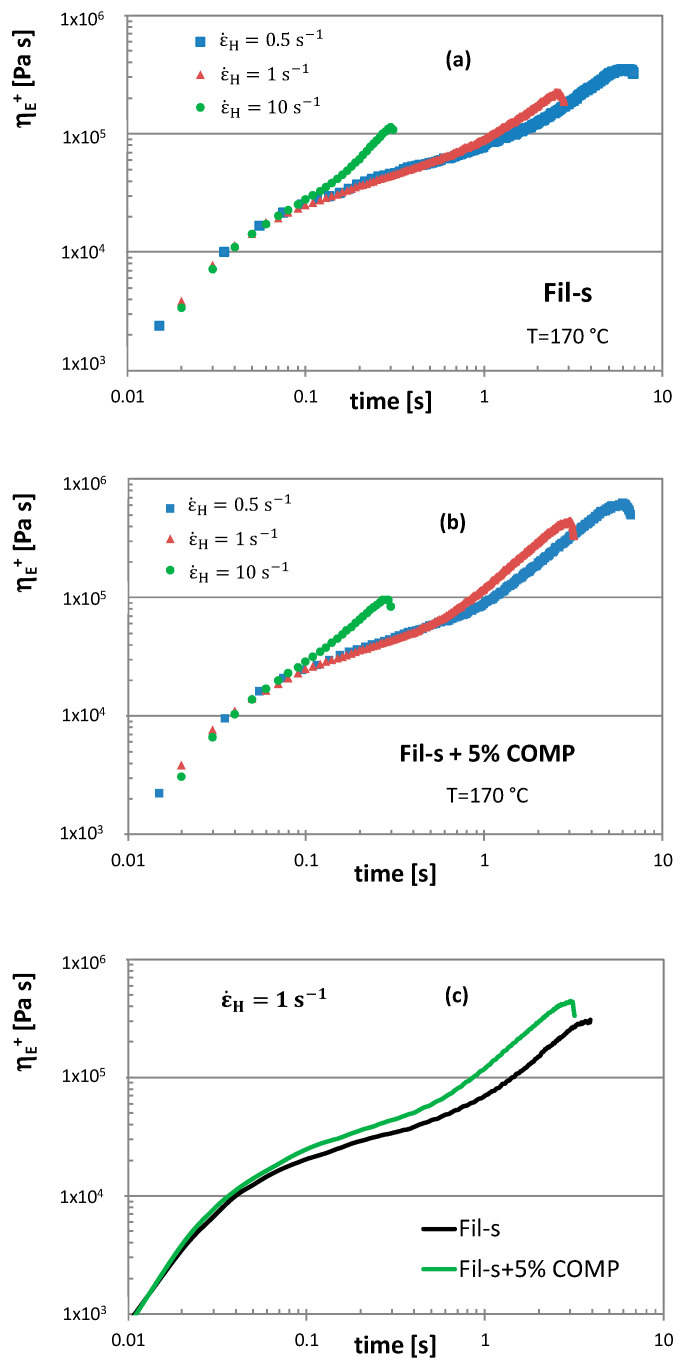
The tensile stress growth coefficient, η_E_^+^, as a function of time at 170 °C and at various Hencky strain rates, ${\dot{\varepsilon }}_{H}$, for: (**a**) neat Fil-s, (**b**) compatibilized Fil-s and (**c**) both samples at ${\dot{\varepsilon }}_{H}\;$ = 1 s^−1^.

The extensional rheological behaviour is also of some concern with regards to the tendency for the melt to deform or sag during the cooling stage of pipes processing [34]. Therefore, transient extensional tests were carried out on both the neat and compatibilized Fil-s and the resulting tensile stress growth coefficient (η_E_^+^) plots, as a function of time, are reported in Figure 6 at different Hencky strain rates (${\dot{\varepsilon }}_{H}$).

Analysing the graphs in Figure 6 it is possible to distinguish two different regions: a linear behaviour at short times, defined as linear extensional region, and a rapid upward deviation from the linearity at long times, denoted as strain hardening region. This latter phenomenon occurs in the molten recyclates due to the presence of long chain branching (specifically in the LDPE fraction of Fil-s), which makes a local obstruction for the stretching of the macromolecules in the direction of deformation [35,36]. It can be also seen that the beginning of strain hardening for both the Fil-s systems depends on ${\dot{\varepsilon }}_{H}$ and starts earlier with increasing the Henchy strain rate. Moreover, comparing the tensile stress growth coefficient plots for the neat Fil-s and the compatibilized one at a fixed ${\dot{\varepsilon }}_{H}$, a more pronounced strain hardening behaviour can be observed for the sample Fil-s + 5% COMP (Figure 6c), evidencing its better resistance to stretching deformations. This outcome is perfectly coherent with the shear rheological results (Figure 5), further supporting the assumption of a higher degree of branching and/or crosslinking of the recyclate due to the addition of the compatibilizer.

### 3.3. Physical-Mechanical Properties of Fil-s Based Systems According with ASTM D3350 Standard

The materials’ specifications, the design and the particular application of polyethylene pipes are regulated by a series of standards and codes, which allow their correct use. In particular, for the characterization of all Fil-s-based systems developed in this study, we referred to ASTM D3350 standard, where six properties are defined as crucial during the service life of polyethylene pipes. Each property corresponds to a “cell” and for each cell there are a number of “classes”, identifying a certain range of values for each specific characteristic. In the present work, we focused on the determination of some of these properties, i.e., density, melt flow index, flexural modulus and tensile strength at yield, not only for Fil-s as such, but also for Fil-s batches submitted to a washing or compatibilizing step in order to improve the recyclate performances.

Successive research activities will concern the measurements of the other two properties, as required by ASTM3350 standard, namely the slow crack growth resistance and the hydrostatic strength. The first one is important for the evaluation of pipes durability and the second one is fundamental for pipes applications concerning the transport of pressurized fluids.

**Figure 7:**
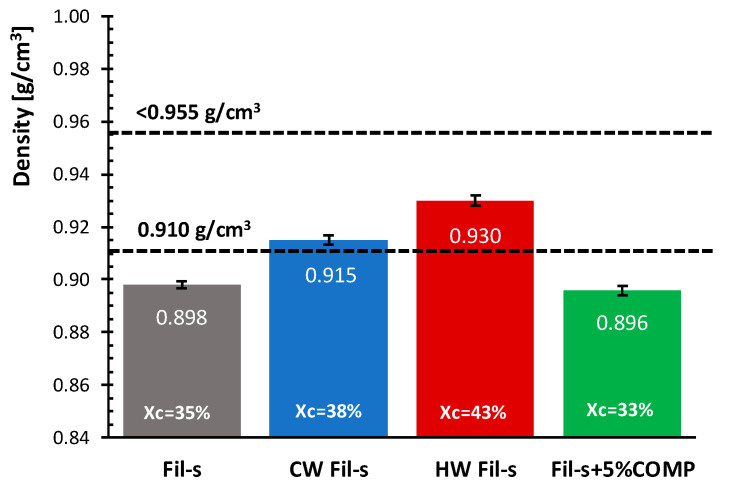
Comparison of the density (ρ) values for the analysed Fil-s based systems. The data relative to the first and last “ρ class”, as defined in ASTM D3350 standard, are also reported.

Density (ρ) affects many of the final performances of polyethylene pipes [37]. In particular, properties such as hardness, tensile strength, stiffness and resistance to chemical agents improve with the increasing of polymer density. However, while small increases in density result in higher load-bearing capability for the pipe, this is achieved at the expense of toughness and slow crack growth resistance [37]. The density values for the analysed Fil-s based systems are reported in Figure 7.

By comparing the density data of the unwashed and washed Fil-s samples, a higher density is observed for these latter, which coherently correlates with an increase in the crystallinity degree for the recyclate following the washing treatments. On the other hand, the addition of the compatibilizer does not involve a significant change in Fil-s density, in agreement with the DSC thermal results, which highlight only a slight reduction in the crystallinity degree for the compatibilized recyclate.

Moreover, by correlating the density values of the recycled systems with the ones corresponding to the first and last “ρ class” reported in the ASTM D3350 standard, it can be evidenced that the washed Fil-s fall within this range, while the density data for the unwashed Fil-s and the compatibilized one are close to the lower limit of this interval.

**Figure 8:**
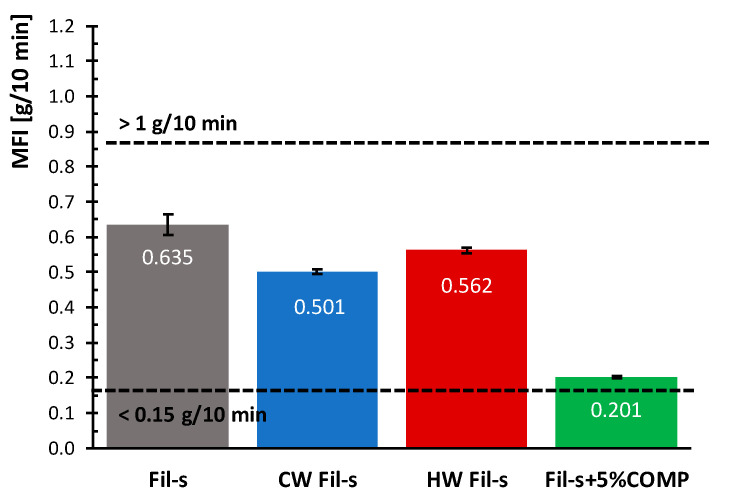
Comparison of the melt flow index (MFI) values for the analysed Fil-s based systems. The data relative to the first and last “MFI class”, as defined in ASTM D3350 standard, are also reported.

The successive property we measured was the Melt Flow Index (MFI), that can be used as a rough indicator of the molecular weight of a polymer. The melt index technique represents an inexpensive means of comparing, in a relative manner, the molecular weight of PEs having similar structure. In particular, resins with a relatively high MFI will have a low to medium molecular weight, which generally tends to compromise the long-term properties of the pipes. Conversely, polymers with a high molecular weight will be characterized by a lower MFI. From this relationship, it is possible to associate changes in melt index with changes in physical properties of the material. However, it is worthy to point out that MFI alone must not be used as a definitive indicator of molecular weight, because variations in polymer structure can affect both molecular weight and melt index. The melt flow index values for all the studied Fil-s systems are compared in Figure 8.

Coherently with the trends of the complex viscosity plots (Figure 2), the MFI value of the unwashed Fil-s is higher than the ones measured for both the CW and HW Fil-s. As already stated, this outcome can be attributed to the effect on viscosity (and therefore on MFI) of the removal from the washed samples of low molecular weight substances, which act as plasticizers in the polymer matrix. However, a slight difference between the MFI values of the washed specimens can also be observed, with a lower MFI for the CW Fil-s. Even if in this study it was verified that the hot washing treatment is able to remove more effectively low molecular weight substances from Fil-s, compared with the cold washing process, further experimental investigations are needed to define in detail which kinds of contaminants were removed. This can affect differently the viscosity and MFI values of CW and HW Fil-s samples.

The addition of the compatibilizer inside Fil-s determines a significant decrease of the MFI parameter. The occurrence of cross-linking and/or branching phenomena among the constituents (main polyolefinic components, polar organic and inorganic contaminants) of the compatibilized Fil-s causes the shift from the prevalent plastic behaviour of the recyclate towards a more rubber-like one [38].

Moreover, the MFI values, corresponding to the first and last class of this parameter as defined by ASTM D3350 standard, have been reported in Figure 8. It can be observed that all Fil-s based systems fall within this range, in particular, the compatibilized Fil-s sample shows flow properties potentially suitable for more demanding applications in the piping sector. In fact, it combines a low MFI value, which can be correlated with a high molecular weight, and so with good long-term performances of the material, with an adequate processability. This behaviour is typical of resins with bimodal molecular weight distribution, generally used for advanced pipes’ applications. However, further experimental investigations, such as creep tests and GPC measurements, are necessary to assess this potentiality in the case of the compatibilized Fil-s.

**Figure 9:**
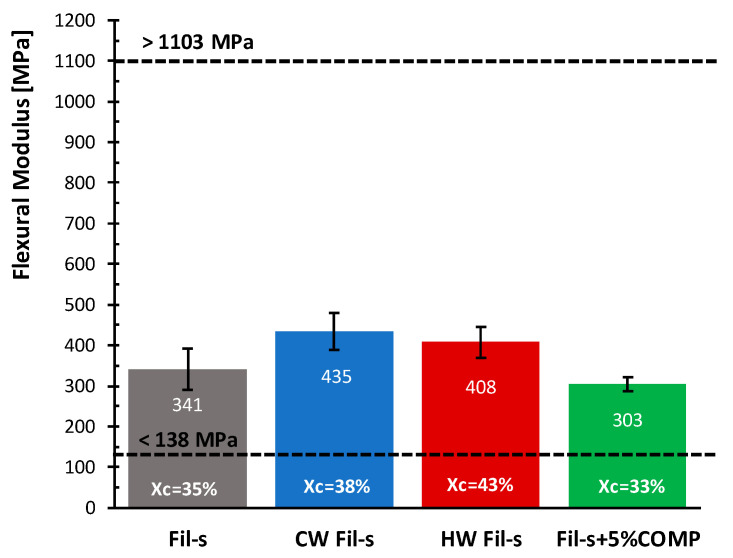
Comparison of the flexural moduli for the analysed Fil-s based systems. The data relative to the first and last “Flexural Modulus class”, as defined in ASTM D3350 standard, are also reported.

Another property, reported by ASTM D3350 standard, is the flexural modulus that is referred as the secant modulus calculated at 2% strain, since most of the polyethylene resins, used for pipes production, does not experience a failure during a bending test. The flexural modulus values for all Fil-s based systems analysed are compared in Figure 9.

A discrete increment in the flexural modulus can be observed for both the washed Fil-s specimens respect to the unwashed recyclate, equal to about 27% for CW Fil-s and 20% for HW Fil-s. In agreement with the spectroscopic (Figure 1), thermal (Table 2) and rheological (Figure 2) results reported in the previous paragraph, the removal of low molecular weight substances, following the washing processes, resulted in a moderate stiffening of the recyclate, which can also be attributed to the increase in the crystallinity degree of the washed samples.

Regarding the compatibilized Fil-s, a slight decrease of the flexural modulus respect to the neat recyclate can be deduced, accordingly with a small decrement of the crystallinity degree, as reported in Figure 9.

Finally, all Fil-s based systems fall within the range of flexural modulus limit values, as specified by ASTM D3350 standard, although none of them exceed the maximum value reported.

A mechanical characterization in tensile mode was also performed for all the recyclate systems under investigation and the main results are reported in Table 4.

**Figure 10:**
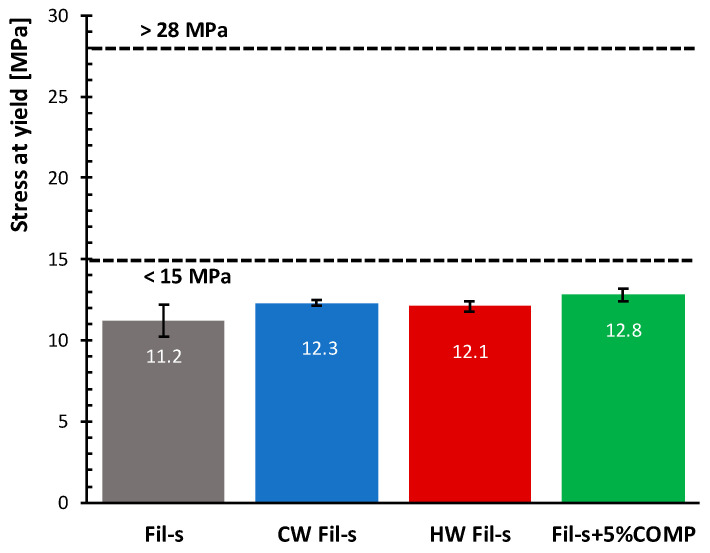
Comparison of the yield stress values for the analysed Fil-s based systems. The data relative to the first and last “σ_Y_ classes”, as defined in ASTM D3350 standard, are also reported.

On the basis of the obtained data, no significant differences are observed for the stress at yield values of the different Fil-s based systems. In particular, all the samples analysed show σ_Y_ values of 15–25% lower compared to the minimum value (15 MPa) of the interval reported in Figure 10, following the specifications of ASTM 3350 standard for this property.

For what concern the Young’s modulus (Table 4), both the washed Fil-s specimens show a slight increase in this parameter compared with the unwashed recyclate, in perfect agreement with the thermal, rheological and flexural mechanical results already discussed for CW and HW Fil-s. However, the stiffness increment for these latter samples is associated with a small reduction in the deformation at break, more pronounced for the HW Fil-s (Table 4). The presence of the compatibilizer determines, instead, a remarkable improvement (about 80%) of the ductility of Fil-s, while no significant change of the elastic modulus can be observed. Considering that the polyethylene grades, generally used for pipes production, have elongation at break values from 400% to 800% [37], it is worthy to point out that all the Fil-s based systems analysed fall within this range.

### 3.4. Production and Characterization of Fil-s Based Pipes

The next step of this study involved the production of pipes with the different Fil-s based systems, using a pilot scale plant. As extensively discussed in our previous work [28], micrometric zeolites were successfully used as innovative moisture adsorbents during Fil-s extrusion to produce simple rigid objects, such as ribbons. In the present study, the desiccant function of zeolites was tested during pipes extrusion of Fil-s, which requires more demanding flow properties compared with the processing of non-hollow and thicker products.

Therefore, the pipes were produced from both the Fil-s based systems previously dried in a vacuum oven and the same not-dehumidified materials (whose moisture contents are reported in Table 2), which were added with 2% by weight of zeolite 4A, this latter working as desiccant during the pipes’ extrusion.

**Figure 11:**
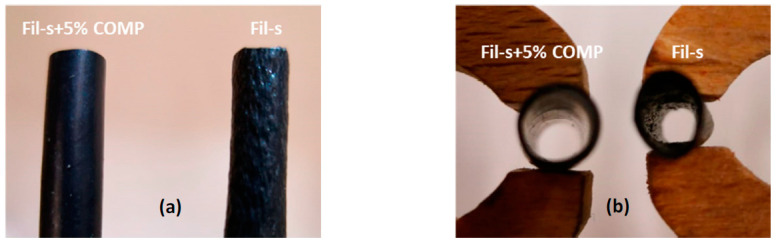
Images of the pipes obtained from Fil-s as such and the compatibilized one: (**a**) the external surface; (**b**) the section.

With all these Fil-s based systems it was possible to obtain pipes with an external diameter of about 6 mm and a thickness of 0.6 mm, even if the neat Fil-s showed some instabilities and ruptures during the extrusion. Conversely, the pipes production with the compatibilized Fil-s resulted stable and continuous, allowing to obtain products without macroscopic defects (Figure 11), as it was expected on the basis of the rheological behaviour, both in shear and extensional mode, of this recyclate sample (Figure 5 and Figure 6).

The produced pipes were then submitted to thermal and mechanical characterization. In particular, the main parameter determined by the compression tests (according to ASTM D2412 standard) was the so-called pipe stiffness, an index of the pipe resistance to crushing. In other words, this property represents the pipe strength to a vertical deformation, caused by an external load along a diametrical plane and it was calculated for all Fil-s based systems at 5 and 10% of deflection. As reported in Table 5, the same trends in the pipe stiffness values can be observed for the pipes obtained from both the heat-dried Fil-s granules and from the not-dehumidified ones, added with the zeolite.

In particular, regarding the pipes produced from the heat-dried Fil-s systems, both the washed specimens show higher pipe stiffness values respect to the unwashed recyclate, with a more pronounced increment (about 65%) in the case of the HW Fil-s, while for the compatibilized Fil-s, the increase in pipe stiffness is less significant and equal to 15%.

The improved mechanical performances of the pipes, made of the compatibilized Fil-s, can be first correlated with the better dimensional uniformity (especially regarding the pipes’ thickness) of these specimens compared with the neat Fil-s pipes. Moreover, in the case of the washed recyclates their enhanced pipe stiffness values can be attributed to the higher crystallinity degrees (X_cPE_, for the PE fraction of these samples) respect to the unwashed Fil-s (Table 6), even if these differences are less pronounced than the ones measured for the same samples extruded as ribbons (Table 3). This can be probably attributed to the different cooling conditions, which occur for the two types of final products.

Although in the absence of zeolite all the undried Fil-s-based systems cannot be extruded as pipes, the addition of this desiccant does not allow to obtain the same performances of the corresponding heat-dried specimens.

In particular, by comparing the stiffness values for the pipes produced from the recyclate systems with the zeolite, the worst performance can be observed for the neat Fil-s, since it shows an evident instability during the pipes extrusion. Conversely, with the compatibilized and washed recyclates the process resulted more stable and continuous. This translates into significant enhancements in pipe stiffness (about 130%) for both the washed Fil-s samples compared with the neat recyclate. Also in the case of the compatibilized Fil-s, the increase in the pipe stiffness is particularly consistent (ca. 68%).

## 4. Conclusions

This study concerned with the possible application of Fil-s, a challenging plastic material coming from the mechanical recycling of post-consumer flexible packaging, in the piping sector. In particular, the research activity focused not only on Fil-s as such, but also on the systems obtained from this recyclate after proper upgrading steps, so to align the material quality with the product requirements. To this purpose, two Fil-s batches, which were submitted to a cold (CW Fil-s) or a hot (HW Fil-s) washing treatment, were analyzed, together with a compatibilized Fil-s sample, obtained by the addition of an experimental additive functionalized with maleic anhydride.

All these Fil-s based systems were characterized according to ASTM D3350 standard, obtaining for each of them a reference technical datasheet that identifies the values for some key properties (specifically density, melt flow index, flexural modulus and tensile strength at yield) associated with the piping performances. The reported results highlight the positive effect of the washing processes, particularly at hot conditions, on properties such as density and flexural modulus, which increased compared with the unwashed Fil-s, due to the removal of low weight contaminants from the recyclate.

On the other hand, the addition of the compatibilizing agent positively affected Fil-s processability by pipe extrusion, as evidenced by the rheological properties (both in shear and extensional mode), allowing to conduct a more stable and continuous process and to obtain pipes without macroscopic defects. In turn, this results in pipe stiffness values higher for the compatibilized specimens than the neat Fil-s, despite a higher crystallinity degree of this latter. However, the best mechanical performance was exhibited by the HW Fil-s with an increase in pipe stiffness equal to about 65% respect to the unwashed sample.

In conclusion, based on the results of this study, a Fil-s batch, first submitted to a hot washing treatment and then compounded with the selected compatibilizing agent, might be a valuable candidate for applications in the piping sector. We will address this issue in our future work, moreover in order to complete the set of key properties for applications in the piping sector (according to ASTM D3350 standard), the slow crack growth resistance and the hydrostatic strength will be also measured for the different Fil-s based systems.

## Figures and Tables

**Table 1:** Main characteristics of zeolites 4A.

Zeolite Name	Structural Type	Cation	Si/Al Ratio	Pores Size [Å]	Particle Size [µm]	Water Uptake after Infinite Time [%]
4A	A type	Na^+^	1.23	3.8	6.50	27.5

**Table 2:** Moisture contents of heat-dried and not-dehumidified Fil-s based systems.

Pellets	Moisture Content [ppm H_2_O]	
	Heat-Dried	Conditioned at 25°C and RH = 75%
Fil-s	270	2500
CW Fil-s	250	1800
HW Fil-s	220	1400
Fil-s + 5%COMP	300	2050

**Table 3:** Main thermal parameters, measured by the second heating scan, for the unwashed, Cold Washed and Hot Washed Fil-s samples.

Sample	T_mPE,1_ (°C)	T_mPE,2_ (°C)	^a^ ΔH_mPE_ (J/g)	X_c,PE_ (%)	T_mPP_ (°C)	^a^ ΔH_mPP_ (J/g)
Fil-s	110	123	102	35%	161	62.2
CW Fil-s	112	124	111	38%	161	47.6
HW Fil-s	112	124	125	43%	161	45.4

^a^ The melting enthalpies (ΔH_mPE_ and ΔH_mPP_) were normalized respect to the relative polyethylene (95 wt%) and polypropylene (5 wt%) contents of Fil-s.

**Table 4:** Mechanical properties (E, Young’s modulus; σ_Y_, ε_Y_, stress and strain at yield; σ_B_, ε_B_, stress and strain at break) for all the investigated Fil-s based systems.

Samples	E [MPa]	σ_Y_ [MPa]	ε_Y_ [%]	σ_B_ [MPa]	ε_B_ [%]
Fil-s	290 ± 15	11.2 ± 1.0	24.5 ± 2.6	10.08 ± 1.0	570 ± 90
CW Fil-s	330 ± 10	12.3 ± 0.2	17. 3 ± 0.4	10.26 ± 0.39	450 ± 140
HW Fil-s	320 ± 15	12.1 ± 0.3	18.1 ± 0.5	10.18 ± 0.25	310 ± 130
Fil-s + 5% COMP	287 ± 8	12.8 ± 0.4	22.0 ± 1.7	12.2 ± 0.4	1030 ± 70

**Table 5:** Pipe stiffness values of heat-dried and not-dehumidified Fil-s based systems.

Treatment	Pipes	Pipe Stiffness [MPa] at Deflection = 5%	Pipe Stiffness [MPa] at Deflection = 10%
Heat-dried	Fil-s	1.80 ± 0.09	1.47 ± 0.05
	CW Fil-s	2.77 ± 0.22	2.50 ± 0.17
	HW Fil-s	2.98 ± 0.41	2.49 ± 0.17
	Fil-s + 5% COMP	2.07 ± 0.14	1.96 ± 0.09
Undried	Fil-s + 2% 4A	0.80 ± 0.12	0.68 ± 0.08
	CW Fil-s + 2% 4A	1.88 ± 0.27	1.60 ± 0.13
	HW Fil-s + 2% 4A	1.85 ± 0.17	1.83 ± 0.08
	(Fil-s + 5% COMP) + 2% 4A	1.34 ± 0.19	1.41 ± 0.14

**Table 6:** Main thermal parameters, measured by second heating scan, for Fil-s based pipes.

Pipes	Tm_PE,1_ (°C)	Tm_PE,2_ (°C)	^a^ ΔHm_PE_ (J/g)	X_c,PE_ (%)	Tm_PP_ (°C)
Fil-s	111	124	110	38%	162
CW Fil-s	111	124	116	40%	161
HW Fil-s	112	124	125	43%	161
Fil-s + 5%COMP	111	124	99	34%	162

^a^ The melting enthalpies (ΔH_mPE_) were normalized respect to the relative polyethylene (95 wt%) content of Fil-s.

## Data Availability

The data presented in this study are available on request from the corresponding author.
